# Prevalence and associated factors of undernutrition among pregnant women visiting ANC clinics in Silte zone, Southern Ethiopia

**DOI:** 10.1186/s12884-020-03404-x

**Published:** 2020-11-19

**Authors:** Mohammed Muze, Mubarek Yesse, Shemsu Kedir, Abdilmejid Mustefa

**Affiliations:** Department of Nursing, Collage of Medicine and Health Science, Werabe University, Werabe, Ethiopia

**Keywords:** Maternal malnutrition, Undernutrition, Pregnant women

## Abstract

**Background:**

Maternal undernutrition is highly prevalent in underdeveloped countries. Hence, this study was intended to determine the prevalence and associated factors of undernutrition among pregnant women visiting ANC clinics in Silte Zone.

**Method:**

*Facility-based* cross-sectional study was conducted from July to January 2019. Systematic random sampling technique was used to select 422 study participants from 11 randomly selected health facilities. Data was collected by using a structured-interviewer administered questionnaire. Mid upper arm circumference (MUAC) was measured by standard non stretchable MUAC tape. Data was entered into a computer using Epi data 3.1 and edited, cleaned, and analyzed using SPSS version 20. Both bivariate and multiple logistic regression analyses were employed to identify factors associated with maternal undernutrition.

**Result:**

In this study, the overall prevalence of undernutrition among study subjects was 21.8%. Age greater than 31 years of women (AOR = 0.15; 95% CI: 0.03, 0.93), Birth intervals > 2 years (AOR = 0.18; 95% CI: 0.04, 0.76), good nutritional knowledge (AOR = 0.34; 95% CI: 0.17, 0.67), and having no dietary change as a result of current pregnancy AOR = 6.02; 95% CI: 2.99, 12.14) were significantly associated with undernutrition.

**Conclusions:**

The prevalence of undernutrition among pregnant women was 21.8%. Current estimate is lower than previously reported in the study area but higher than reported in developed country. Age of women, Birth intervals, and Dietary change as a result of current pregnancy and Nutrition knowledge were important risk factors/ predictors of undernutrition (MUAC < 23 cm). Interventions targeting maternal nutrition education and child spacing with giving special emphasis to adolescent pregnant women are recommended.

## Background

Undernutrition and poor health from avoidable causes excessively affect the health of millions of people in developing countries [1]. Women and young children are the most affected [2]. Child and maternal undernutrition is responsible for approximately 3.5 million deaths of under-five age children [3]. Undernutrition during pregnancy physiologically demanding period would result in adverse pregnancy outcomes [4] Undernutrition makes the women more susceptible to diseases, more risk of having miscarriages, and give birth to low weight baby whose survival is at risk [3, 5].

Nutritional status during pregnancy is directly linked to intrauterine growth retardation (IUGR) and birth weight [6–8]. Babies with fetal growth restriction are at increased risk of death throughout infancy [9]. Globally, about 23.8% of newborns affected by IUGR were born every year. Overall, about 75% of all affected newborns are born in developing countries [10].

Previous studies showed that maternal undernutrition is one of the risk factors for under-five undernutrition [11, 12] which remains an alarming rate: wasting still impacts the lives of too many young children in developing countries [13–15]. Immune dysfunction in malnourished mothers has a causal effect on infant’s nutritional status. Reduced transfer of protective maternal immune factors increased exposure to pathogenic microbes and pro-inflammatory mediators confer an elevated metabolic cost on developing infants and contributes to the development of undernutrition [16].

Apart from the serious consequences of a person’s health, the economy is also affected by undernutrition [17]. High prevalence of undernutrition hampers economic development and preserves poverty both directly, through a loss of productivity due to poor physical condition, and indirectly, through poor cognitive function and learning deficits [18]. Undernourished children are more likely to become short adults, to have a poor educational achievement, and to give birth to smaller infants [19].

In developing nations including Ethiopia, burden of maternal and child undernutrition is very high. The prevalence of maternal undernutrition in Africa including Ethiopia found between 11 and 36% [20–27]. The most acceptable explanation for this wide variation is likely to be the fact that contextual factors are major determinants of maternal undernutrition.

Previous studies found that age at first marriage, educational status, Poor nutritional knowledge, dietary practice, and marital status were discovered as risk factors for undernutrition among pregnant women [28–30]. Risk factors for undernutrition might not be the same across different regions due to differences in socioeconomic characteristics, culture, ethnicity, and geographical location. Ethiopia is a multicultural country.

In the year 2025, the WHO has planned to reduce anemia by 50% and low birth-weight by 30% [14]. Ethiopian government launches a national nutrition program. Maternal and child nutrition is one of its targets that need updated data on the nutritional profile of pregnant women which is essential to develop effective intervention strategies to prevent maternal and child undernutrition. Furthermore, there is limited data on prevalence and factors associated with maternal undernutrition in the study area particularly the southern region of Ethiopia. Therefore, maternal undernutrition has to be understood in a specific context to develop effective and tailored interventions. Therefore, this study was aimed to determine the prevalence and associated factors of undernutrition among pregnant women visiting ANC clinics in Silte Zone.

## Methods

### Study design, period, and setting

Institution-based cross-sectional study was conducted to assess the prevalence and associated factors of undernutrition among pregnant women visiting ANC follow up clinics of health facilities. It was conducted from July to January 2019 in Silte Zone. It is one of fourteen Zones of the southern region of Ethiopia and found 172 km away from Addis Ababa (the capital city of Ethiopia). The administrative center of the zone is Werabe town. This zone consists of 3 administration towns, and 10 rural weredas. Based on the last Census conducted by the central statistical agency of Ethiopia, in 2018 the Zone has an estimated population of 1,017,557 of whom 34,510 were expected pregnant women. About 13% of the total population is urban inhabitants. Also there are 4 Hospitals and 33 Health centers.

### Study subjects

The source population of this study was all pregnant women who were attending ANC (Antenatal care) follow up in the health facilities found in Silte Zone. The study population was selected pregnant women who were attending ANC follow up in the health facilities found in Silte Zone during the study period. Women who were seriously ill, those who had hand deformity, and those who had revisit during the study period were excluded.

### Sample size determination and sampling technique

The sample size required for the study was computed using a single population proportion formula. Considering the absence of similar study conducted in similar context as per our search effort, we took *p*-value of 50, 5% marginal error, and 95% confidence interval were used to obtain the maximum sample size. With the addition of 10% non-response rate, the initial sample size was increased from 384 to 422. In the study area, antenatal care service is provided in all health care facilities. To increase the generalizability of the results, health facilities were stratified based on the type of the health facility, 1 hospital from the four hospitals and 10 health centers from the 33 health centers were selected randomly. The calculated sample size was allocated proportionately to each stratum. According to the health facilities report, on average, 15–30 pregnant women visit the ANC daily and 2066 pregnant women have been enrolled in ANC of selected health facilities. Forty two from Tora primary hospital and 380 from 10 health centers were selected using a systematic random sampling technique at interval of k = 5. Of the first five pregnant women, the 3rd woman was randomly selected by using a lottery method. Accordingly, every 5th pregnant women were chosen based on their visiting order until the sample size was met. Participants were approached after receiving service.

### Measurements and data collection tools

An interviewer-administered questionnaire was used to collect the data. Socio-demographic factors (such as Age, Marital status, Residence, Occupation, maternal educational status, religion, and husband educational status), Obstetric history of respondents, nutrition knowledge, dietary practice, and anthropometric data were collected. Sociodemographic items were adapted from the Ethiopian demographic and health survey (EDHS 2016) [31]. Knowledge questions were adopted from the Food and Agricultural Organization of the United Nation (FAO) knowledge, attitude, and practice survey guideline [32]. Nutritional Nutrition knowledge of women consists of micronutrient supplements, benefits of folic acid supplements, health risks for low–birth–weight babies, and birth interval effects on women’s nutrition. Five items measuring nutritional knowledge were scored on a dichotomous scale (0 = “does not know” response, 1 = “knows”). For each question, a correct answer was coded as 1 and an incorrect answer as 0. The total score was a maximum of 5. Mean is used as a cut off value to categorize nutritional status. Dietary change as a result of current pregnancy was assessed by a single item “Do you change a diet as a result of current pregnancy?” [33].

Measurement of middle upper arm circumference (MUAC) was used to determine the nutritional status of pregnant women. Mid upper arm circumference of the left arm was measured triplicate using a non-stretchable standard MUAC tape to the nearest 0.1 cm with no clothing on the arm. The mean of triplicate measurement was taken. Undernourished considered when Pregnant women’s MUAC was < 23 cm and ≥ 23 cm was considered well-nourished [23, 34, 35]. Meal frequency was considered to be recommended (adequate) when respondents take an additional meal (> 3 meals per day) as a result of the current pregnancy [36].

### Data quality control

Earlier to the data collection, data collectors and supervisors were trained. The instrument was pretested in 5% of the sample size. Pretest was conducted on individuals with similar characteristics of the study population who were not a part of the actual study. Based on the pretested results, the instrument was modified and changed. Trained supervisors supervised the data collection and check the completeness of the questionnaire on a daily base. Furthermore, principal investigators carefully cleaned and entered collected data into a computer. During the entry of data, double-entry verification by Epidata 3.1 was applied.

### Data processing and analysis

Data were entered into a computer using Epi data version 3.1, cleaned, and analyzed using SPSS version 20. Bivariable and multivariable logistic regression was used to determine the degree of association between independent and dependent variables. Variables with *p*-value of < 0.25 in Bivariable logistic regression analysis were candidates for multiple logistic regression analysis. In multivariable logistic regression analysis, those variables with a *p*-value ≤0.05 with adjusted odds ratio interval exclude one was considered as statistically significant.

## Results

### Socio-demographic characteristics of respondents

From the total 422 pregnant women, 417 were participated in this study, making a response rate of 98.8%. The mean age of the participants was 26.6 ± 5 years and 39.1% of the participants were in age between 26 and 30 years. About three fourth (72.3%) of the participants were housewives/unemployed. From the total, 198 (47.5%) participants had low educational status. Majority (76.7%) of women were rural residents. [Table 1 summarizes the socio-demographic characteristics of respondents).

**Table 1:** Sociodemographic characteristics of respondents in Silte zone, Southern Ethiopia, 2019, (*n* = 417)

Variables		Frequency	Percent
**Age of the women in years**	15–20	70	16.8
	21–25	117	28.1
	26–30	163	39.1
	31–35	52	12.5
	› = 36	15	3.6
	Total	417	100
**Religion**	Orthodox	10	2.4
	Muslim	405	97.1
	Others	2	0.5
	Total	417	100
**Marital status**	Single	23	5.5
	Married	393	94.2
	Divorced	1	.2
	Total	417	100
**Women’s education status**	Illiterate	198	47.5
	None formal education	92	22.1
	Primary level (1–8 grade)	100	24.0
	Secondary level and above	27	6.5
	Total	417	100
**Women’s occupational status**	Unemployed	350	83.9
	Employed	67	16.1
	Total	417	100
**Husband’s employment**	unemployed	288	73.3
	Government employee	80	20.4
	Private employee	25	6.4
	Total	393	100
**Residency**	Urban	97	23.3
	Rural	320	76.7
	Total	417	100

### Obstetric history of respondents

The average number of children per woman was 3 ± 2. The mean birth interval between current pregnancy and previous last pregnancy is 2.7 ± 1. More than half (66.4%) of the pregnant women were in their third trimester of pregnancy. The majority 216(56.1%) of the women reported that they had > = 5 family size. One hundred thirty (44.2%) of women gave birth with 3 year interval.

### Nutrition knowledge, maternal dietary practice, and prevalence of under nutrition

This study found 91 (21.8%) of study subjects were undernourished. The majority 285(68.3%) of them had recommended meal frequency. Two hundred sixty four (63.3%) of women changed diet as a result of the current pregnancy. About half 213(51.1%) of women had good nutritional knowledge.

### Factors associated with undernutrition

A multivariable analysis in a form of logistic regression was employed to identify the associated factors of undernutrition among pregnant women. The analyses rest on two outcomes of the nutritional status of pregnant women: whether they are undernourished (MUAC< 23 cm) or not (MUAC≥23 cm). On Bivariable analysis, Age of women, Birth interval, Daily meal frequency, Nutrition knowledge, Dietary change as a result of current pregnancy were significantly associated with undernutrition. Variables with *p*-value of < 0.25 in the Bivariable logistic regression analysis were entered into multivariate logistic regression analysis. In multivariate analysis, Age greater than 31 years of women (AOR = 0.15; 95% CI: 0.03, 0.93), Birth intervals > 2 years (AOR = 0.18; 95% CI: 0.04, 0.76), good nutritional knowledge (AOR = 0.34; 95% CI: 0.17, 0.67), and having no dietary change as a result of current pregnancy AOR = 6.02; 95% CI: 2.99, 12.14) were significantly associated with undernutrition (Table 2).

**Table 2:** Factors associated with nutritional status of pregnant women in Silte Zone, Southern Ethiopia, 2019, (*n* = 417)

Variables		Nutritional status(n)		*P* value	Crude odds ratio (95% C.I.)	*P* value	Adjusted odds ratio (95% C.I.)
		Well-nourished (> = 23 cm)	Under nourished (< 23 cm)				
Age of the women in years	15–20	49	21		1		1
	21–25	94	23	0.109	0.57 (0.29,1.13)	0.102	0.26 (0.05,1.31)
	26–30	125	38	0.283	0.71 (0.38,1.33)	0.400	0.52 (0.11,2.4)
	31–35	46	6	0.019	0.3 (0.11,0.82)	0.041	0.15 (0.03,0.93)
	> = 36	12	3	0.44	0.58 (0.15,2.28)	0.506	0.49 (0.06,4.01)
Employment status of husband	Unemployed	222	66		1		1
	Government employee	61	19	0.876	1.05 (0.58,1.88)	0.905	1.06 (0.44,2.56)
	Private employee	23	2	0.101	0.29 (0.07,1.27)	0.106	0.15 (0.07,1.5)
Birth interval	One year	10	9		1		1
	Two years	86	19	0.00	0.25 (.009,0.69)	0.003	0.10 (0.02,0.44)
	Three years	101	29	0.02	0.32 (0.12,0.86)	0.020	0.18 (0.04,0.76)
	Four years	23	5	0.03	0.24 (0.06,0.91)	0.009	0.10 (0.02,0.57)
	Five and above years	11	1	0.04	0.10 (0.01,0.95)	0.045	0.07 (0.01,0.95)
Gestational age	1st trimester	5	3		1		1
	2nd trimester	111	21	0.133	0.32 (0.07,1.42)	0.505	0.42 (0.034,5.3)
	3rd trimester	210	67	0.396	0.53 (0.12,2.28)	0.717	0.64 (0.06,7.39)
Daily meal frequency	> 3	90	42		1		1
	≤3	236	49	0.001	0.45 (0.28,0.72)	0.739	0.87 (0.39,1.94)
Nutrition knowledge	Poor	142	62		1		1
	Good	184	29	0.000	0.36 (0.22,0.59)	0.002	0.34 (0.17,0.67)
Dietary change as result of current pregnancy	Yes	230	34		1		1
	No	96	57	0.000	4.02 (2.47,6.54	0.000	6.02 (2.99,12.14)

n refers to frequency

## Discussion

The purpose of this study was to assess the prevalence and associated factors of undernutrition among pregnant women visiting the ANC clinics in Silte Zone, southern Ethiopia. The current study found one in every five women was undernutrition. Age of the women, Dietary change as a result of current pregnancy, Birth intervals, and Nutrition knowledge were significant determinants of undernutrition. The current study found 21.8% (MUAC< 23) prevalence rate of undernutrition. Our result is consistent with those of the other three studies carried out in other parts of Ethiopia 19.8, 19.5 and 24% [23, 24, 30]. It is also consistent with findings of studies done in south Sudan, Kenya and a systemic review in Africa reported 18.9,19.3 and 23.5% respectively [20, 37, 38] .

On the other hand, our finding is higher than those of studies conducted in other three areas of Ethiopia and Madagascar reported 16.2, 14.4, 9.2, and 9% respectively [22, 29, 39, 40]. Our finding is also higher than the study reported in china where 11.8% of pregnant women were undernourished [28].

The variation might be due to differences in MUAC cut of value, and the socio-culture distinctions between Ethiopia and the other counties. The present study finding is lower than those of studies done in other areas of Ethiopia reported 72, 34, 31.8, 31.4, 30.3, and 28.6% [26, 27, 41–44]. The prevalence of undernutrition in this study is also lower than 32% reported in Bangladesh [45]. The current study showed that the prevalence of undernutrition among pregnant in the country is decreasing as compared to earlier studies. The discrepancy might be due to effective nutritional intervention. Another possible reason may be due to a difference in season of studies conducted. In Ethiopia, household food security varies with season. Furthermore, the variation may be due to geographical variation.

Nutrition knowledge of pregnant women was significantly associated with maternal undernutrition. It was negatively associated with maternal undernutrition. The odds of undernutrition among pregnant women with poor nutritional knowledge were higher than their counterparts. This finding is similar to a previous study [23]. This could be possibly due to that poor nutritional knowledge about nutrition usually results in poor dietary intake leading to undernutrition.

In the current study, the age of pregnant mothers was found to be negatively associated with the undernutrition of pregnant women. Pregnant women who are at age of between 31 and 35 years have decreased the risk of undernutrition by 15% when compared to pregnant women age less than 31 years. Consistent findings are reported in other studies [21, 22, 39, 46]. This could be possibly due to early age women often have a lack of awareness about their own health and nutritional status that leads to poor nutritional status. Adolescent women have no or little power in decision- making about food distribution in the household is another reason that leads to poor nutritional status. It might also be due to less experience of maternity and related complications among adolescent women.

Dietary change as a result of the current pregnancy was significantly associated with maternal undernutrition. Women who did not improve their eating habits as a result of current pregnancy had 6 times higher odds of having undernutrition when compared to their counterparts. Similar findings were reported in other studies [24, 47].

Interval between current and just previous pregnancy found inversely associated with the undernutrition of pregnant women. Pregnant women who had birth interval greater than 2 years had a lesser risk of undernutrition compared to those who had a birth interval of fewer 2 years. A similar finding was reported in a study conducted in Bangladesh [48]. There are many reasons to suspect that a short birth interval could adversely affect nutritional status of the mother or the child. For the mother, a short birth interval may give her insufficient time to recover from the nutritional burden of pregnancy [49].

### Limitations of the study

The study was facility based and the study subjects may not represent the general population.

## Conclusions

The prevalence of undernutrition among pregnant women was 21.8% current estimate is lower than previously reported in the study area, but higher than reported in developed countries. Age of the women, their birth interval, dietary change as a result of current pregnancy, and knowledge on nutrition were factors significantly associated with maternal undernutrition in the current study. Interventions targeting maternal nutrition education, and child spacing with giving special emphasis to adolescent pregnant women are recommended.

## Data Availability

Data sets used and analyzed during the current study are available from the corresponding author on reasonable request.

## Abbreviations

ANC: Antenatal care
EDHS: Ethiopia demographic and health survey
FMOH: Federal ministry of health
IUGR: Intrauterine growth retardation
MCH: Mother and child health
MOH: Ministry of health
NGO: Non-governmental Organization
SNNPR: Southern Nations, nationalities, and peoples Region
SPSS: Statistical package for social science
WHO: World health Organization

## References

[1] Adrianne B (2008). Nutrition and health in developing countries.

[2] Uthman O, Aremu O (2008). Malnutrition among women in sub-Saharan Africa: rural-urban disparity. Rural Remote Health.

[3] Black RE, Allen LH, Bhutta ZA, Caulfield LE, de Onis M, Ezzati M (2008). Maternal and child undernutrition: Global and regional exposures and health consequences. Lancet.

[4] Bhutta ZA, Das JK, Rizvi A (2013). al e. evidence-based interventions for improvement of maternal and child nutrition: what can be done and at what cost?. Lancet..

[5] United nations administrative committee on coordination and subcommittee on nutrition. Symposium Report on a Nutrition Policy Discussion Paper on Women and Nutr. Geneva; 1990.

[6] Wu G, Bazer FW, Cudd TA, Meininger CJ, Spencer TE (2004). Maternal nutrition and fetal development. J Nutr.

[7] Lechtig A, Yarbrough C, Delgado H, Habicht JP, Martorell R, Klein RE (1975). Influence of maternal nutrition on birth weight. Am J Clin Nutr.

[8] Imdad A, Bhutta Z (2012). Maternal nutrition and birth outcomes: effect of balanced protein-energy supplementation. Paediatr Perinat Epidemiol.

[9] Black RE, Victora CG, Walker SP, Bhutta ZA, Christian P, De Onis M (2013). Maternal and child undernutrition and overweight in low-income and middle-income countries. Lancet.

[10] de Onis M, Blössner M, Villar J (1998). Levels and patterns of intrauterine growth retardation in developing countries. Eur J Clin Nutr.

[11] Farah AM, Endris BS, Gebreyesus SH. Maternal undernutrition as proxy indicators of their offspring’s undernutrition: evidence from 2011 Ethiopia demographic and health survey. BMC Nutr. 2019;5(17). 10.1186/s40795-019-0281-z. 10.1186/s40795-019-0281-z PMC7050883 32153930

[12] Gewa CA, Oguttu M, Yandell NS (2012). Maternal nutrition in rural Kenya: health and socio-demographic determinants and its association with child nutrition. Matern Child Nutr.

[13] Unicef. Malnutrition rates remain alarming: stunting is declining too slowly while wasting still impacts the lives of far too many young children. 2019;2020.

[14] World health organization. WHO. In: Child malnutrition: WHO; 2020. https://www.who.int/news-room/fact-sheets/detail/malnutrition.

[15] World Banck. Nutrition country profiles. 2020.

[16] Prendergast AJ, Humphrey JH (2014). The stunting syndrome in developing countries. Paediatr Int Child Health.

[17] Bagriansky J, Champa N, Pak K, Whitney S, Laillou A (2014). The economic consequences of malnutrition in Cambodia, more than 400 million US dollar lost annually. Asia Pac J Clin Nutr.

[18] Lewit EM, Baker LS, Corman H, Shiono PH (1995). The direct cost of low birth weight. Futur Child.

[19] Victora CG, Adair L, Fall C, Hallal PC, Martorell R, Richter L (2008). Maternal and child undernutrition: consequences for adult health and human capital. Lancet.

[20] Desyibelew HD, Dadi AF. Burden and determinants of malnutrition among pregnant women in Africa: A systematic review and meta-analysis. PLoS One. 2019;14(9). 10.1371/journal.pone.0221712. 10.1371/journal.pone.0221712 PMC6730925 31490956

[21] Mtumwa A, Paul E, Vuai S (2016). Determinants of undernutrition among women of reproductive age in Tanzania mainland. S Afr J Clin Nutr.

[22] Kumera G, Gedle D, Alebel A, Feyera F, Eshetie S. Undernutrition and its association with socio-demographic, anemia and intestinal parasitic infection among pregnant women attending antenatal care at the University of Gondar Hospital, Northwest Ethiopia. Matern Heal Neonatol Perinatol. 2018;4(18). 10.1186/s40748-018-0087-z. 10.1186/s40748-018-0087-z PMC6134711 30214818

[23] Diddana TZ (2019). Factors associated with dietary practice and nutritional status of pregnant women in Dessie town, northeastern Ethiopia: a community-based cross-sectional study. BMC Pregnancy Childbirth.

[24] Kedir H, Berhane Y, Worku A (2016). Magnitude and determinants of malnutrition among pregnant women in eastern Ethiopia: evidence from rural, community-based setting. Matern Child Nutr.

[25] Yimer M. The prevalence of under nutrition and associated factors among adolescent pregnant women: a narrative review. J Med Care Res Rev. 2020;3(2). 10.15520/mcrr.v3i2.87.

[26] Belete Y, Negga B, Firehiwot M. Under Nutrition and associated factors among adolescent pregnant women in Shashemenne District, West Arsi Zone, Ethiopia: a community-based study. J Nutr Food Sci. 2016;6(1). 10.4172/2155-9600.1000454.

[27] Regassa N, Stoecker BJ (2012). Contextual risk factors for maternal malnutrition in a food-insecure zone in southern Ethiopia. J Biosoc Sci.

[28] Gao H, Stiller CK, Scherbaum V, Biesalski HK, Wang Q, Hormann E (2013). Dietary intake and food habits of pregnant women residing in urban and rural areas of Deyang City, Sichuan Province. China Nutr.

[29] Dadi AF, Desyibelew HD (2019). Undernutrition and its associated factors among pregnant mothers in Gondar town, Northwest Ethiopia. plos One.

[30] Gebre B, Biadgilign S, Taddese Z, Legesse T, Letebo M (2018). Determinants of malnutrition among pregnant and lactating women under humanitarian setting in Ethiopia. BMC Nutr.

[31] Central Statistical Agency [Ethiopia] and ICF International (2016). Ethiopia Demographic and Health Survey. Addis Ababa, Ethiopia, and Calverton.

[32] Yvette Fautsch Macías (2014). Guidelines for assessing nutrition-related Knowledge, Attitudes and Practices.

[33] Oh HK, Kang S, Cho SH, Ju Y, Faye D. Factors influencing nutritional practices among mothers in Dakar, Senegal. PLoS One. 2019;14(2). 10.1371/journal.pone.0211787 PMC6370274 30742655

[34] Tang AM (2016). Determining a Global Mid-Upper Arm Circumference Cutoff to Assess Malnutrition in Pregnant Women.

[35] Alice M (2013). eta'l. use of cutoffs for mid-upper arm circumference (MUAC) as an Indicator or predictor of nutritional and health related outcomes in adolescents and adults: a systematic review.

[36] Institute of Medicine (1990). Nutrition during pregnancy. Part 1: Weight gain and Part II: Nutrient supplements. Committee on Nutritional Status During Pregnancy and Lactation.

[37] Alemayehu A, Gedefaw L, Yemane T, Asres Y (2016). Prevalence, severity, and determinant factors of Anemia among pregnant women in south Sudanese refugees, Pugnido, Western Ethiopia. Anemia.

[38] Willy KJK, Peter C (2016). Dietary diversity, nutrient intake and nutritional status among pregnant women in Laikipia County, Kenya. Int J Health Sci Res.

[39] Dadi AF, Desyibelew HD. Undernutrition and its associated factors among pregnant mothers in Gondar town, Northwest Ethiopia. PLoS One. 2019;14(4). 10.1371/journal.pone.0215305. 10.1371/journal.pone.0215305 PMC6476509 31009475

[40] Ravaoarisoa L, Randriamanantsaina L, Rakotonirina J, Rakotomanga J, Donnen P, Dramaix M (2018). Socioeconomic determinants of malnutrition among mothers in the Amoron’i mania region of Madagascar: a cross-sectional study. BMC Nutr.

[41] Sonko A. Assessment of dietary practice and anthropometric status of pregnant women in Aleta Chuko Woreda Southern Nations, nationalities and people’s™ Region /SNNPR/, Ethiopia: descriptive cross-sectional study. Epidemiol Public Health Rev. 2016;1(1). 10.16966/2471-8211.102.

[42] Mariyam AF, Dibaba B. Epidemiology of malnutrition among pregnant women and associated factors in Central Refit Valley of Ethiopia, 2016. J Nutr Disorders Ther. 2018;8(1). 10.4172/2161-0509.1000222.

[43] Masresha L, Maleda T, Mohammed G (2019). Factors associated with malnutrition among pregnant women and lactating mothers in Miesso health center, Ethiopia. Eur J Midwifery.

[44] Nigatu M, Gebrehiwot TT, Gemeda DH. Household food insecurity, low dietary diversity, and early marriage were predictors for Undernutrition among pregnant women residing in Gambella, Ethiopia. Adv Public Heal Hindawi. 2018:1–10.

[45] Hossain B, Sarwar T, Reja S, Akter MN. Nutritional status of pregnant women in selected rural and urban area of Bangladesh. J Nutr Food Sci. 2013;3(4). 10.4172/2155-9600.1000219.

[46] Serbesa ML, Iffa M, Geleto M (2019). Factors associated with malnutrition among pregnant women and lactating mothers in Miesso health center, Ethiopia. Eur J Midwifery..

[47] Mamta S, Anant N, Sarika P, Mukesh S, Shriram G, Vikrant P (2019). Undernutrition and its association with socio-demographic factors among pregnant women attending tertiary health care hospital in northern Maharashtra: a cross sectional study. Int J Community Med Public Health.

[48] Khan KS, Chien PFW, Khan NB (1998). Nutritional stress of reproduction. A cohort study over two consecutive pregnancies. Acta Obstet Gynecol Scand.

[49] King JC (2003). The risk of maternal nutritional depletion and poor outcomes increases in early or closely spaced pregnancies. J Nutr.

